# Evaluation of the Effect of Orlistatorlistat on Expression of *OCT4, Nanog, SOX2,* and *KLF4* Genes in Colorectal Cancer SW40 Cell Line

**DOI:** 10.31557/APJCP.2021.22.8.2335

**Published:** 2021-08

**Authors:** Mojgan Noroozi Karimabad, Farzad Roostaei, Mehdi Mahmoodi, Mohammad Reza Hajizadeh

**Affiliations:** 1 *Molecular Medicine Research Center, Research Institute of Basic Medical Sciences, Rafsanjan University of Medical Sciences, Rafsanjan, Iran. *; 2 *Department of Clinical Biochemistry, Faculty of Medicine, Rafsanjan University of Medical Sciences, Rafsanjan, Iran. *; 3 *Department of Clinical Biochemistry, Afzalipoor Faculty of Medicine, Kerman University of Medical Sciences, Kerman, Iran. *

**Keywords:** Orlistat, colorectal cancer, gene expression

## Abstract

**Background and Objective:**

Orlistat drug is one of the most criticalanti-obesity drugs that widely used around the world. The aim of this study was evaluation the effect of orlistat on the expression of *OCT4, Nanog, SOX2*, and *KLF4* genes in the colorectal cancer SW40 cell line.

**Materials and Methods:**

SW40 cell line was cultured in DMEM medium contained orlistat for 24h, and cell viability was assessed by MTT assay. The fold changes of expression of *OCT4, NANOG, KLF4,* and* SOX2* at mRNA level against β-actin were determined by real-timePCR. Two-sample t-test and one-way ANOVA were used to compare the mean of expression of different genes in different groups and different concentrations; a significant level of 0.05 was considered in all tests.

**Results:**

Our results showed a significant difference in cell viability, when different doses of Orlistat were used for 24 hour. concentrations of 25 and 100 μM reduce significantly the expression of *OCT4* (P<0.05) and *SOX2* (P<0.05) in the treated group in comparison to control (P<0.05). Also, the mRNA expression of *KLF4 *and *Nanog *was reduced significantly after treatment of SW40 cell lines was performed with 100 μM doses of Orlistat (P<0.05).

**Conclusion:**

It appears that after further studies in animal and human phases, orlistat can be used for the treatment of Colorectal Cancer.

## Introduction

Colorectal cancer is the most common cancer of the digestive tract; it affects the colon, rectum, and appendages (Markowitz and Bertagnolli, 2009; Karimi Zarchi et al., 2011). About 1 million people die each year due to this disease, with the highest incidence rates in North America and Europe and the lowest incidence rates in Asia, Africa, and South America (Karimi Zarchi et al., 2011). The incidence of this disease is 7-9 Per 100,000 in Iran and the death rate is 1.198 per 100,000 (Momeni et al., 2012). The most critical causes of colorectal cancer are genetic and nutritional factors (Ashmore, 2013). The most important treatment options for colorectal cancer include surgery, radiotherapy, and medication such as 5-fluorouracil (De Rosa et al., 2015; Hammond et al., 2016). (Octamer 4) *OCT4, NANOG*, (SRY-related HMG-box gene 2) *SOX2 KLF4* (Krupel-like factor4) genes are transcription factors that control the differentiation and design of embryonic stem cells; however, recent studies have indicated that the expression of these genes is involved in the development of many cancers, including the bladder, liver, breast, and colorectal cancer, pvalue is (P<0.05) (Gillis et al., 2011; Ibrahim et al., 2012; Yin et al., 2013; Nagata et al., 2014; Amaya and Bryan, 2015; Müller et al., 2016) OCT4 function in tumors is due to OCT4 / Tcl1 / Akt pathway. The high expression of OCT4 results in an increase in Tcl1 and increased expression of this oncogene, which reduces the phosphorylation of Akt, while Akt phosphoryl acts as a stimulant for induction of planned death (apoptosis) ((Wang et al., 2010; Asadi et al., 2011). The functional mechanism of the* SOX2* gene for breast cancer suggests that the gene’s contribution to tumorigenicity is facilitated by passing the cell from step G1 to S. Although the *KLF4* gene was initially recognized as anti-epitope and anti-apoptosis in differentiated cells, recent studies have shown that the expression of this gene increases in many cancers such as gastric, intestinal, bladder, and kidney cancer, and it is closely associated with poor prognosis (El-Karim et al., 2013; Feizi et al., 2013; Schoenhals et al., 2013). Like OCT4, NANOG is increased in many cancers; it is regulated by the *OCT4*-ending gene due to the effect of *OCT4* and *SOX2*, and activation of* p53 *causes suppressing transcription. The impact of this gene on cancer is due to the alignment of cancer cells with the immune system, inhibition of apoptotic-associated genes, and facilitating cellular transfusion from regulatory sites (Wang et al., 2013). Orlistat, as a weight-loss agent, is a synthetic derivative of lipostativin that affects lipid metabolism. This medicine has been shown to inhibit pancreatic and stomach lipase, which is the result of preventing triglyceride deficiency and inhibiting the absorption of triglyceride (Martin et al., 2013); the inhibition of FAS (Fatty Acid Synthesis) is another functin of orlistat. Recently, the relationship between this enzyme and the incidence of cancer has been closely noted (Kridel et al., 2004). Some side effects of orlistat include bloating, osteoarthritis, increased stool volume, abdominal cramping, and reduced-fat intake of vitamins (García et al., 2013).

The limited number of studies have been conducted, and the results are sometimes contradictory Regarding the association of orlistat with cancer. According to a study by Nairooz and Garcia which was conducted on rats receiving orlistat, this medicine, as an anti-obesity drug in the colon, could induce cell proliferation, severe changes in colon cribs, and an increase in the amount of Aberrant Cryptococcus (ACF) (Nairooz et al.; Garcia et al., 2006). ACFs are a group of quasi-tubular glands that, unlike normal cells, are resistant to apoptosis, cover the entire colon and rectum, and are one of the first changes leading to cancer in the colon (Takayama et al., 2005). Another study by Cioccoloni et al. suggests that orlistat may increase the risk of gastrointestinal cancers, such as colorectal cancer, by decreasing DNA repair (Cioccoloni et al., 2015). However, the Orsolin et al. study found that orlistat can not mutate and ultimately generate cancer in body cells (Orsolin et al., 2012). In addition, other studies suggest that orlistat not only has no role in developing cancer, but also has an anti-cancer effect (Chuang et al., 2011; Azadbakht et al., 2015); the effect of this drug on the expression of the FAS enzyme is one of the proposed mechanisms. Increasing the expression of FAS in colorectal cancer represents a poor prognosis in people who are affected, because the FAS enzyme plays an important role in the regulation of pro-apoptotic proteins and cellular processes, such as DNA repair (increased repair), and therefore its expression increases the resistance to FAS drugs, such as orlistat, therefore enhancing apoptosis in cancer cells (Ogino et al., 2007; Wu et al., 2014). In addition, this medicine can stop the progression of the cell cycle in cancer cells, increase apoptosis, and decrease the expression of the oncogene *Her2 / neu* expression in these cells (Menendez et al., 2005). Based on the above-mentioned points, since orlistat is safely prescribed for obese people for weight loss, and given the fact that there are previous contradictory results about the mechanism of its molecular effects in the development of colorectal cancer; the present case study was conducted to examines the possible consequences of medication, for the first time, on the expression of *OCT4, NANOG, SOX2* and *KLF4* genes in cancer.

## Materials and Methods

The SW40 cells (colorectal cancer) was purchased from the National Cell Bank of Iran (NCBI, Tehran, Iran). SW40 cell line was prepared in 25 cm flasks containing DMEM medium, 10% fetal calf serum (FBS), 100 u/ml penicillin, and 100 μg/ml streptomycin and incubated in CO_2_ 37°C. For testing, the cells are separated from the flask by trypsin-EDTA and centrifuged at about 1,100 rpm for 7 minutes. The cellular deposition is carried out in suspension at 1 cc of DMEM medium.

*MTT assay*

1x10^4^ cells per each well were seeded onto a 96-well plate at a final volume of 200 μL. After cells treated by 25, 50 and 75, 100 and 120 μg / mL orlistat , the supernatant was replaced with 200 μL of warm RPMI 1640 (without phenol red). Following the add 10 μL of 5 mg/ml MTT to each well, plates were incubated at 37^o^C for 3.5 hours in the dark room, until the development of a purple precipitate was visible under the light microscope. Then 100 μL of DMSO (Dimethyl sulfoxide) was added to each well, and after 15 min, the absorbance was read at 570 (Karimabad et al., 2017; Akbarpoor et al., 2020).

Then, to determine the effect of concentrations of 25 and 100 mM of orlistat on the expression of *OCT4, NANOG, SOX2,* and *KLF4* genes, 2×l0^6 ^cells was cultured in 25 cm flasks. The incubation time for the cells was 24 hours. Then, the supernatant was removed, and the environments containing concentrations of 25 and 100 μg / ml of orlistat were added. The incubation time of the treatments was 24 hours. Controlling the SW40 cell line contains no orlistat. All of the experiments were repeated three times. Extracting mRNA from studied cells and synthesize cDNA

The fidelity of extracted RNA was examined by electrophoresis 1% agarose and further staining with DNA Green Viewer™ and three bands with the Gel Doc was seen from each RNA sample.se, 

DNase was used to remove DNA from; MMLV cDNA enzyme was used to synthesize strands from all mRNA molecules present in the specimen. OligoT Primer and Revert AID First Strand cDNA Synthesis (Fermantas) kit will be used to build cDNAs. Then, a Reverse primer and a Forward primer, as well as the Taq DNA polymerase enzyme, ATCG nucleotides and appropriate buffering media, and the Siber-green fluorescence color, DNA synthesized from the above genes are reproduced in Applied Biosystems thermocycler (US) (Rezai et al., 2018).

*Real-Time PCR*

The gene expression of *OCT4, NANOG, SOX2*, and *KLF4* was detected by real time PCR. To achieve this, the deserved of synthesized cDNA was taken at this step and well mixed with a master mix contains DNA Taq polymerase. With the help of specific primers (Table 1), *OCT4, NANOG, SOX2*, and *KLF4* expression were determined, employing RT PCR and using a BIO-Rad Real-Time system (Bio-Rad Company, USA). The RT-PCR temperature conditions were 95°C for 30 seconds for initial extinction, 40 cycles each containing 95°C for 5 seconds for exhaustion, 60°C for 30 seconds for annealing and extension. The β-actin was used as a reference gene, and the expression of target genes relative to its expression. Relative expression was calculated using the comparative 2^-ΔΔCt^ formula (Noroozi et al., 2019).

*Stastictical analysis method*

To statistically analyze the data, SPSS software (version 21) was applied. All experiments were performed three times in each sample, and all of the obtained results were presented as the mean value of those three. Two-sample t-test and one-way ANOVA were used to compare the mean of expression of different genes in different groups and different concentrations; a significant level of 0.05 was considered in all tests.

## Results

**Figure 1:**
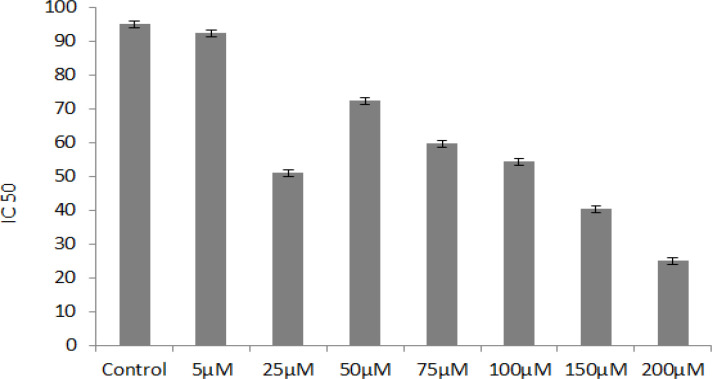
MTT Results of the Effect of Orilsat on SW40 Colorcetal Cancer Cells

Results after MTT (Sun et al., 2015), it was found that only two concentrations of 25 mM And 100 mM were considered as IC50among various concentrations of 5 mM, 25 mM, 50 mM, 75 mM, 100 mM, 125 mM, and 150 mM of orlistat material (Figure 1). 

Expression of Nanog, *OCT4, SOX2*, and *KLF4* genes in the present study, the effects of concentrations of 25 and 100 μM of orlistat on the expression of Nanog, *OCT4, SOX2*, and *KLF4* genes was investigated.

**Figure 2:**
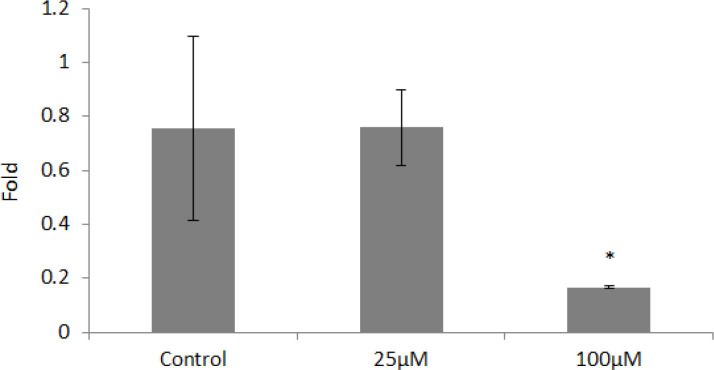
*KLF4* Gene Expression in Different Concentrations of Orlistat

Based on statistical tests, 100 μm concentration decreased the expression of the *KLF4* gene significantly (p <0.05), while 25 μm concentration did not have a significant effect on expression of this gene (p <0.05) (Figure 2).

**Figure 3:**
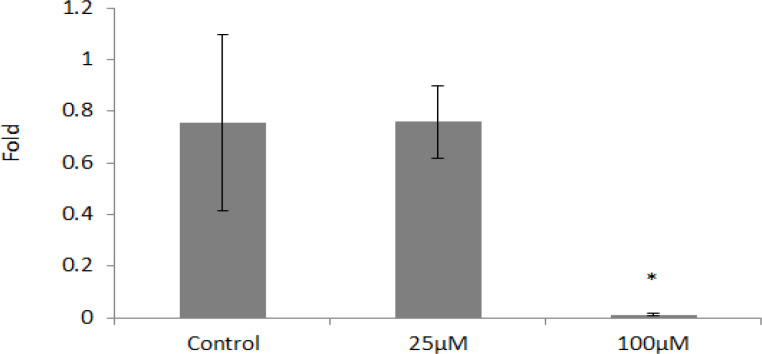
*Nanog* Gene Expression in Different Concentrations of Orlistat

The results of statistical tests showed that although 25 μm concentrations of orlistat reduced the expression of the Nanog gene expression, this expression reduction was not statistically significant (p <0.05), while 100 μm concentrations of orlistat decreased the expression of this gene is significantly (p <0.05) (Figure 3).

**Figure 4:**
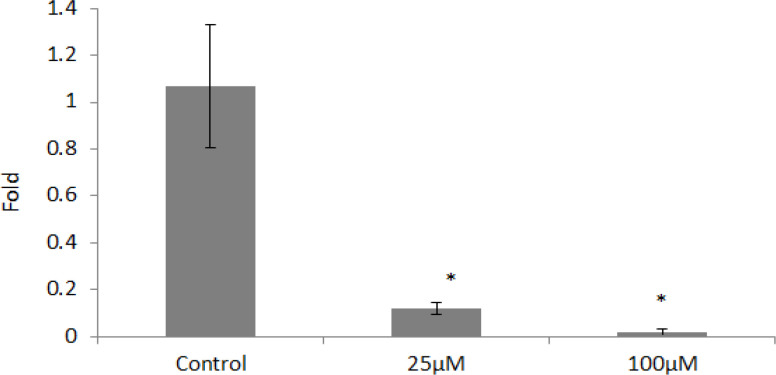
*SOX2 *Gene Expression in Different Concentrations of Orlistat

**Figure 5:**
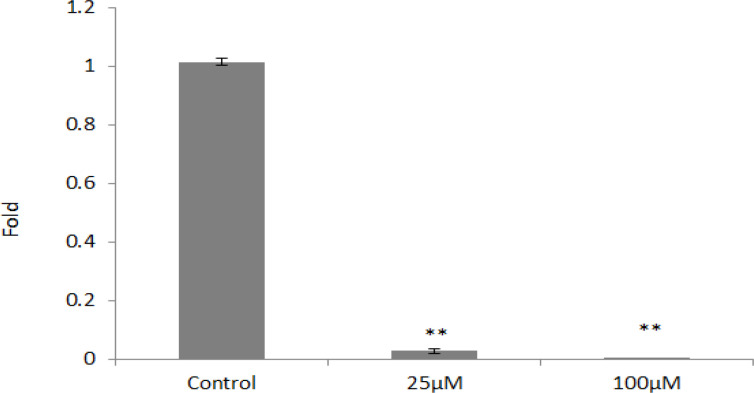
*OCT4* Gene Expression in Different Concentrations of Orlistat

*SOX2* was the other gene assessed in the present study; based on statistical tests concentrations of 25 and 100 μm reduced the expression of this gene significantly (p <0.05) (Figure 4). As shown in Figure 5, both 25 and 100 μm doses of orlistat decreased the* OCT4* gene expression significantly. (p <0.05) (Figure 5).

**Table 1:** Sequence of Primers Used in the Present Study

Target gene	Primer sequence
*OCT4*	CGCAAGCCCTCATTTCAC
	CATCACCTCCACCACCTG
*NANOG*	GCAACGAGTGCAAGGCAC
	CGAACGAGAGAGACAGCA
*KLF4*	GCCAGACAGACTCCGTTAGG
	GCAGGCTAAGTTCTGGCGG
*SOX2*	CCGATTAGCTTGACGTGG
	CGAGGCTTATGCCATTG
*B-Actin*	CACACCTTCTACAATGAGC
	ATAGCACAGCCTGGATAG

## Discussion

In the present study, the effects of concentrations of 25 and 100 μM of orlistat on the expression of *Nanog, OCT4,* *SOX2*, and *KLF4* genes were investigated using the Real-time technique. The results of this study showed that doses of 25 and 100 μm resulted in a significant reduction of *Oct4* gene expression. In addition, a 100 μm dose of this medication reduces the expression of *NANOG, SOX2,* and *KLF4* significantly. *OCT4, NANOG, SOX2*, and *KLF4* genes are genes that code transcription factors of the same name; these factors have important implications for the differentiation and growth of stem cells; however, recent studies indicate that the expression of these genes increases in many cancers, such as gastrointestinal cancers. The results of Saigusa et al. study show that the expression of *Sox2 *and *Oct4* increases in patients with colorectal cancer; in addition, the increased expression of these genes in these individuals increases with relapse of the disease and metastasis after chemotherapy (Saigusa et al., 2009). The results of Meng (2010) study showed that the expression of the *Nanog* gene is not only increased in colorectal cancer, but also its expression is also associated with a poor prognosis and progression of the disease (Meng, 2010). In addition to the above studies, Leng et al., (2013) research showed that the expression of the *KLF4* gene is increased significantly in colorectal cancer cells. Effective factors on the expression of this gene can be effective in treating colorectal cancer. 

Orlistat, as a weight-loss agent, is a synthetic derivative of lipostativin that affects lipid metabolism. This medication has been shown to inhibit pancreatic and gastric lysis and the FAS enzyme, which prevents the inhibition of triglyceride uptake and fatty acid synthesis (Martin et al., 2013; Kridel et al., 2004). Considering that orlistat is one of the most widely used medicines in the world, many studies have already been conducted on the association of this drug with cancer. Menendez et al, study, which, was conducted to investigate the effect of orlistat on breast cancer, showed that in addition to affecting the expression of the *Her/neu* gene as one of the most critical oncogenes, this medicine stops the cellulite and increasing apoptosis in cancer cells. Another study by Kridel et al. indicated that orlistat, in addition to inhibiting the growth and proliferation of cancer cells, induces apoptosis in prostate cancers (Kridel et al., 2004). In another study by Bhargava-Shah on breast cancer, the results indicated that orlistat could be associated with microRNAs to improve chemotherapy(Bhargava-Shah et al., 2016). The results of some studies consistent with the present one in regard to breast cancer, prostate cancer, and ovarian cancer, suggest that orlistat causes suppression of genes involved in carcinogenesis (Gansler et al., 1997; Menendez et al., 2005; Huang et al., 2012; Sadowski et al., 2014). Various studies have shown that orlistat can inhibit the expression of multiple enzymes (Kridel et al., 2004; R Pandey et al., 2012), including *OCT4, NANOG, SOX2,* and *KLF4*. An increase in the expression of the microRNA 145 (miRNA 145) by orlistat seems to be one of the possible mechanisms for reducing the expression of these genes. Several studies have shown that expression of miRNA 145 is one of the most important factors in controlling the expression of, and quenching, factors involved in reprogramming cells such as OCT4 and NANOG during several cancers, such as colorectal damage, and increased expression of the target genes of this miRNA is one of the results of this reduction in expression. Therefore, orlistat may reduce the expression of the gene by increasing the expression of miRNA. (Hamfjord et al., 2012; Cui et al., 2014).

However, the present study examines the new dimensions of the therapeutic effects of orlistat on colorectal cancer and its effect on the expression of some of the most important genes involved in colorectal cancer that have not been taken into consideration before; however, like other studies, the present research had some limitations such as monitoring the expression of the genes studied and the lack of evaluation of the effect of this substance on some genes such as tumors of the suppressors.

The results of this study showed that orlistat reduces the expression of *OCT4, NANOG, SOX2,* and *KLF4* genes. According to the results of the study and the use of orlistat, it is suggested for later studies to assess the effects of this medicine on different cancers as well as other genes, such as neurotransmitter suppressors.

## Author Contribution Statement

Study Design, Mohammad Reza Hajizadeh; Data Collection, Farzad Roostaei; Data Interpretation , Mehdi Mahmoodi; Manuscript Preparation, Mojgan Noroozi Karimabad Mohammad Reza Hajizadeh Mehdi Mahmoodi; Literature Search, Mojgan Noroozi Karimabad, Farzad Roostaei; Funds Collection, Mohammad Reza Hajizadeh

## Data Availability

All data and materials as well as software applications support our published claims and comply with field standards.
